# Role of cervical fascia flap in reducing the incidence of pharyngocutaneous fistula after total laryngectomy

**DOI:** 10.1007/s00405-026-10179-y

**Published:** 2026-04-06

**Authors:** Donia Usama Hamza, Ahmed Musaad Abd El-Fattah, Ahmed Hemdan, Hemmat Baz, Hisham Atef Ebada

**Affiliations:** 1 Department of Otolaryngology – Head and Neck Surgery, Faculty of Medicine, Mansoura University, Mansoura, Egypt; 2 Phoniatric Unit, Faculty of Medicine, Mansoura University, Mansoura, Egypt

**Keywords:** Laryngeal carcinoma, Total laryngectomy, Pharyngocutaneous fistula, Swallowing, Cervical fascia

## Abstract

**Objectives:**

The aim of the current randomized clinical trial is to evaluate the efficacy of the superiorly based cervical fascia flap in reducing the incidence of the PCF after total laryngectomy. Additionally, the functional outcomes in terms of postoperative swallowing functions were evaluated.

**Methods:**

This parallel randomized clinical trial included 50 patients undergoing total laryngectomy, randomly assigned to either a cervical fascia flap group (*n* = 25) or a control group (*n* = 25). In the cervical fascia group, a superiorly based flap from the investing layer of the deep cervical fascia was used to reinforce the pharyngeal closure. The primary outcome was the incidence of PCF. Secondary outcomes included postoperative swallowing function assessed three months postoperatively using the Eating Assessment Tool (EAT-10) and videofluoroscopic swallowing study.

**Results:**

PCF occurred in 2 patients (8%) in the cervical fascia group compared with 8 patients (32%) in the control group, demonstrating a statistically significant reduction (*p* = 0.034). Swallowing outcomes were significantly better in the cervical fascia group, with lower median EAT-10 scores (*p* = 0.001) and more favorable videofluoroscopic findings (*p* = 0.039).

**Conclusion:**

The use of cervical fascia for pharyngeal repair after total laryngectomy is effective in reducing the incidence of pharyngocutaneous fistula. Its main advantages include technical simplicity, cost-effectiveness, and availability within the same surgical field, without the need for additional incisions, prolonged operative time, or donor-site morbidity. Furthermore, this technique is associated with favorable postoperative swallowing outcomes.

## Introduction

Total laryngectomy is considered a definitive treatment for advanced-stage tumors (T3 and T4a) and is also commonly employed as a salvage approach in recurrent disease when nonsurgical modalities, including radiotherapy or chemotherapy, have been unsuccessful [1, 2]. Pharyngocutaneous fistula (PCF) is the most common complication after total laryngectomy [3] with reported incidence in literature varying from 5 to 65% [4–6].

PCF is associated with substantial postoperative morbidity after laryngectomy, as it is linked to extended hospitalization, higher healthcare expenditures, and postponement of adjuvant therapy [7–9]. In addition, this complication may result in life-threatening outcomes, including fatal carotid artery rupture or aspiration pneumonia [8, 10].

Several well-recognized risk factors contribute to the development of PCF, including preoperative irradiation, low hemoglobin levels, and prior tracheotomy. However, surgical technique during pharyngeal closure remains a major factor in the occurrence of PCF [6, 11].

Several strategies have been suggested to decrease the PCF rates, including the application of mechanical staplers in pharyngeal closure [10, 12], the application of biomaterials to promote healing [13, 14], and the routine incorporation of myofascial flaps in patients undergoing salvage laryngectomy [15, 16].

Zorlu et al. [17] proposed a novel technique to reduce the incidence of PCF after laryngectomy, in which a superiorly based fascial flap was fashioned from the superficial layer of the deep cervical fascia overlying the strap muscles and then sutured over the pharyngeal repair line.

This randomized clinical trial was conducted to evaluate the efficacy of the superiorly based cervical fascia flap in decreasing the incidence of PCF after total laryngectomy. Additionally, the functional outcomes in terms of postoperative swallowing functions were evaluated.

## Patients and methods

### Study design and setting

This parallel randomized clinical trial was conducted in the Department of Otorhinolaryngology, Faculty of Medicine, Mansoura University, Egypt, over a 1.5-year period (June 2024 to December 2025). Written informed consents were obtained from enrolled patients. Ethical approval was received from the Mansoura Faculty of Medicine Institutional Research Board (IRB: MS.24.08.2855). The study was retrospectively registered at ClinicalTrials.gov in November 2025 (NCT07241611). This randomized clinical trial was conducted and reported in accordance with the Consolidated Standards of Reporting Trials (CONSORT) guidelines.

### Sample size calculation

An a priori sample size calculation was performed based on the primary outcome of pharyngocutaneous fistula incidence. Based on previously published literature reporting PCF rates of approximately 30–35% following total laryngectomy and assuming a clinically meaningful reduction to 10–15% with the use of cervical fascia reinforcement [17], an absolute risk reduction of 20% was anticipated. With a two-sided alpha level of 0.05 and a statistical power of 80%, a minimum of 24 patients per group was required. Accordingly, a total sample size of 50 patients was planned.

**Fig. 1:**
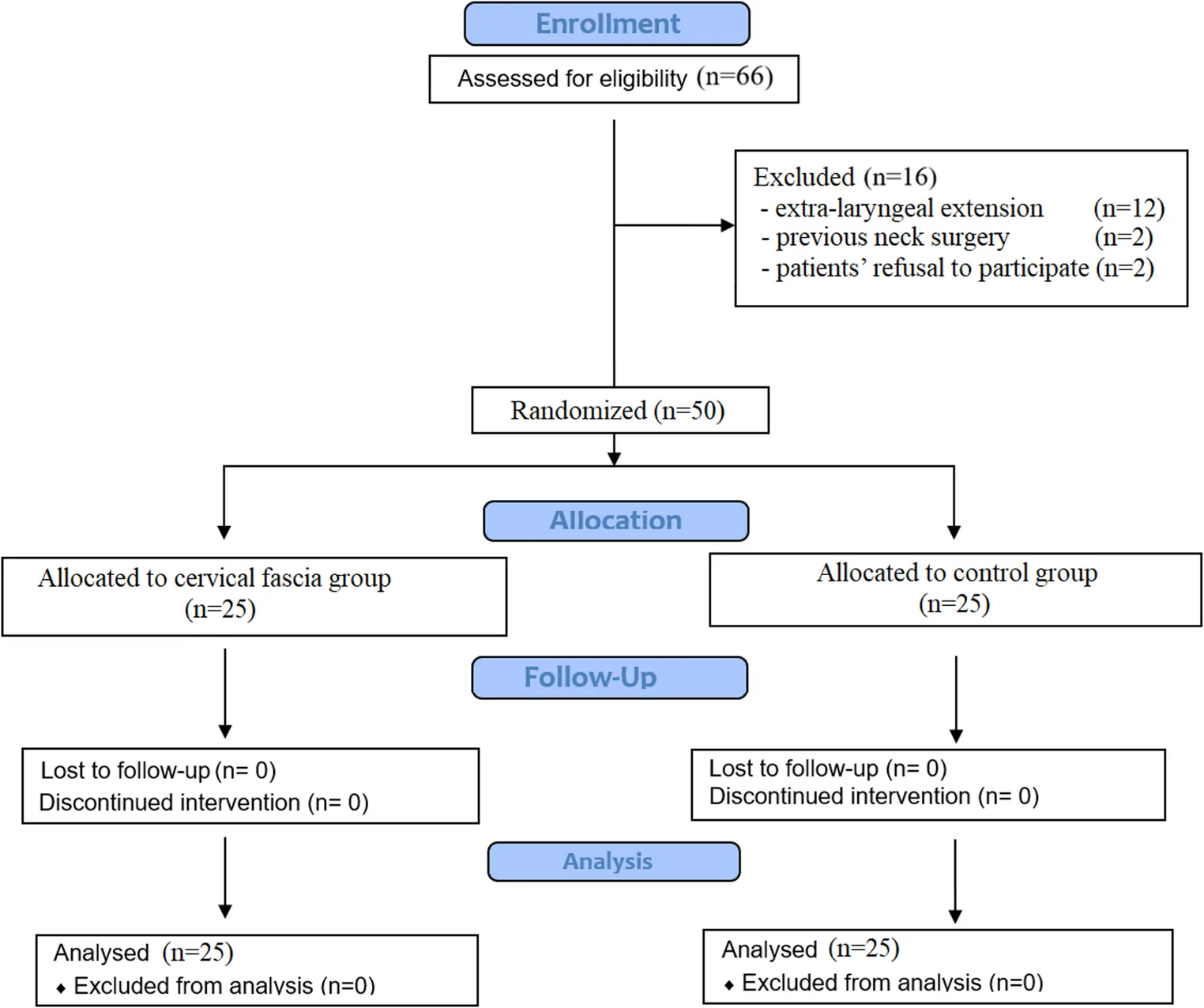
The study flow chart

All individuals who underwent total laryngectomy within the study period (*n* = 66) were initially eligible for inclusion (Fig. 1). Of these, 16 patients were excluded because of extra-laryngeal extension of the tumor (*n* = 12), previous neck surgery (thyroidectomy) (*n* = 2), or refusal to participate in the study (*n* = 2). The remaining 50 patients were included.

#### Randomization, allocation concealment, and blinding

Patients (*n* = 50) were randomly allocated to 2 groups: the cervical fascia group (*n* = 25) and the control group (*n* = 25). Randomization was performed using a computer-generated random sequence created by an independent investigator who was not involved in patient enrollment, surgery, or outcome assessment. Group allocation was concealed using sequentially numbered, sealed envelopes prepared in advance. Each envelope was opened just before total laryngectomy procedure, thereby ensuring allocation concealment and minimizing selection bias.

Assessment of the primary outcome (development of pharyngocutaneous fistula) and secondary outcome (postoperative swallowing function) was performed by clinicians who were blinded to group allocation and were not involved in the surgical procedure or postoperative decision-making.

### Surgical techniques

Total laryngectomy was carried out in all cases using a uniform, standardized surgical approach, and all procedures were performed by the same surgical team. In the cervical fascia group, after the skin incision, and the subplatysmal flap elevation, a superiorly based flap from the investing layer of the deep cervical fascia was fashioned. A transverse incision was made in the investing fascia covering the strap muscles, at the inferior level of the strap muscles before starting laryngectomy.

**Fig. 2:**
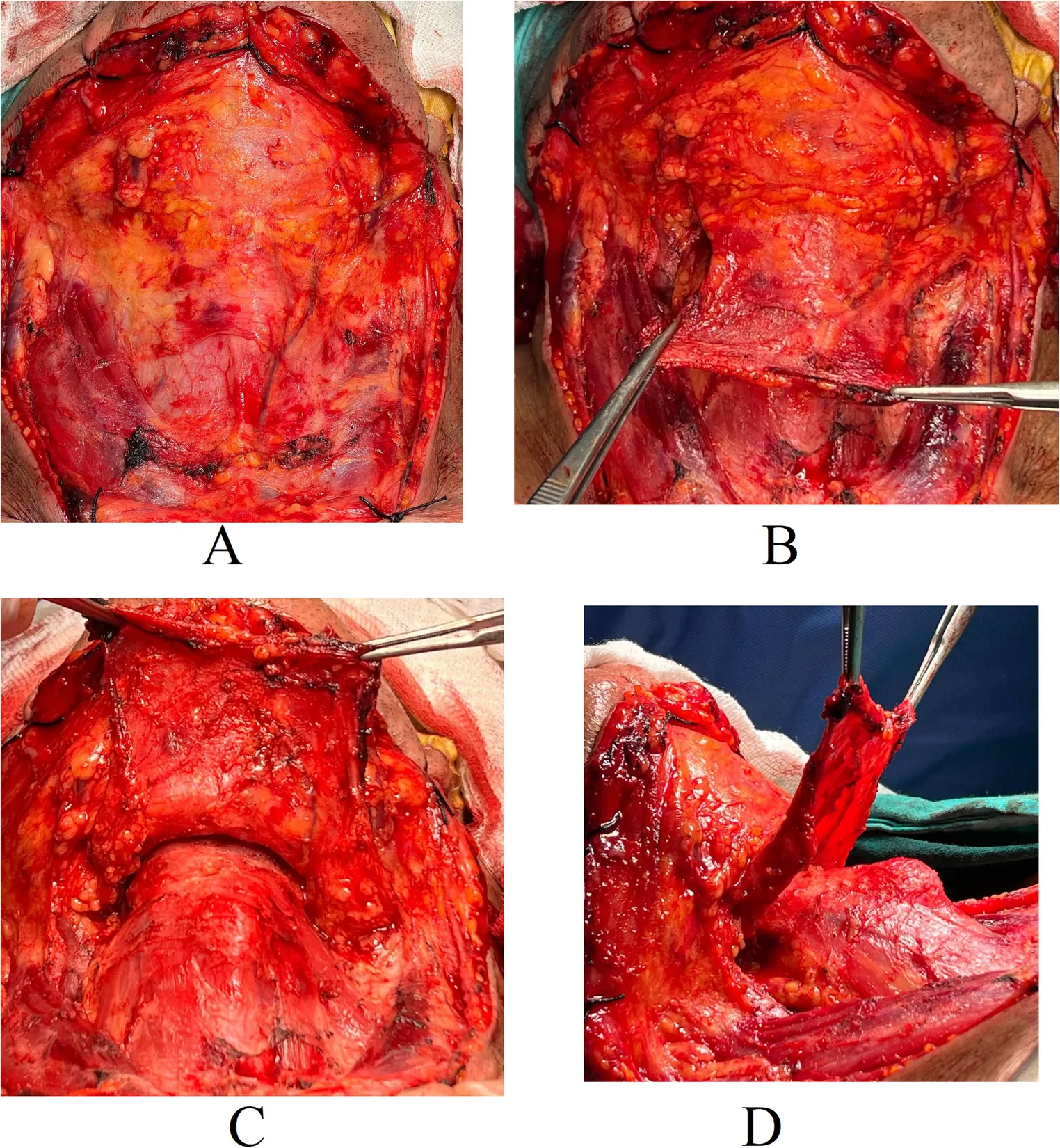
Creation of the cervical fascia flap. **A**: The investing layer in place after elevation of the subplatysmal flaps. **B**: The fascia flap is incised. **C**: The flap is elevated and pedicled superiorly. **D**: Lateral view showing the fascia flap

Afterward, the flap was elevated superiorly in a U-shaped fashion, with the lateral border of the strap muscles forming the lateral border of the flap. The hyoid bone is exposed, and the fascia is dissected to the submental region (Fig. 2).

**Fig. 3:**
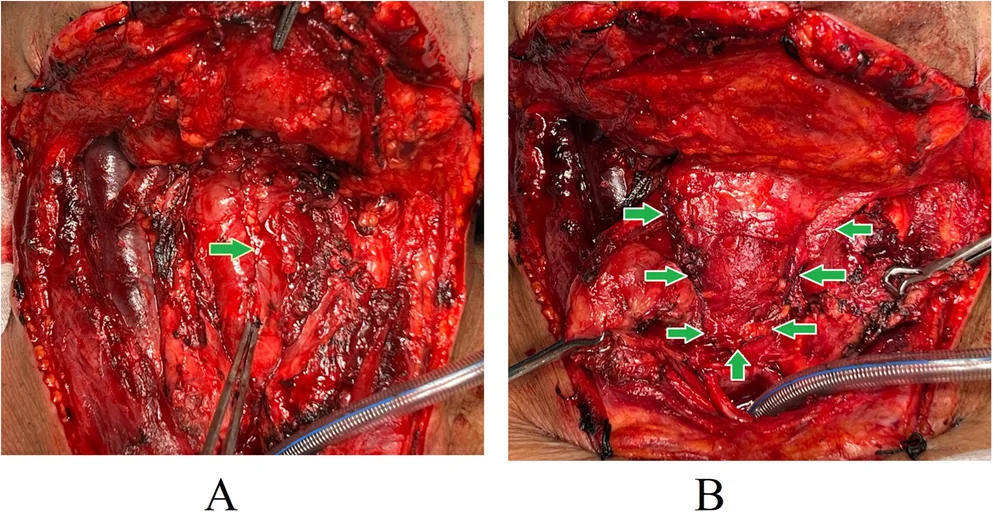
Application of the flap on the pharyngeal repair. **A**: The line of pharyngeal repair (the arrow). **B**: The fascia flap after its fixation over the pharyngeal repair. The arrows point to the borders of the flap

After the laryngectomy is completed, pharyngeal repair was achieved using a continuous inverting suture technique with Vicryl sutures. The fascia was then laid on the pharyngeal closure line and sutured to the underlying tissues with anchoring sutures (Fig. 3). In the cervical fascia group the constrictor muscle was not sutured over the mucosal repair.

In the control group, pharyngeal closure was completed in a two-layer fashion. The pharyngeal mucosa was approximated using a continuous inverting suture technique with Vicryl sutures, followed by reinforcement of the mucosal layer through suturing of the pharyngeal constrictor muscles. Subsequently, a constrictor myotomy was undertaken to reduce the risk of postoperative dysphagia.

### Postoperative care

All patients were administered intravenous antibiotics for one week after surgery. Proton pump inhibitors were also prescribed for 2 weeks. Feeding through a nasogastric tube was commenced on the second postoperative day and continued for a total period of 10 days. Surgical drains were retained for at least three days and were subsequently removed individually once the daily output decreased to 30 mL.

The diagnosis of PCF was primarily based on clinical assessment. Early indicators included wound dehiscence, local erythema, and the presence of serous, sanguineous, or purulent discharge. The diagnosis became definitive with the appearance of frank salivary leakage through a cutaneous defect at or adjacent to the surgical incision.

On the tenth postoperative day, evaluation for fistula formation was performed by asking the patient to swallow sterile water or methylene blue dye, with careful observation for leakage from the wound edges or the presence of subcutaneous fluid collection. In the absence of leakage and clinical signs of pharyngocutaneous fistula, the nasogastric tube was removed, and oral intake was initiated with liquids and semisolid foods. Conversely, in cases where a fistula was detected, the nasogastric tube was left in situ, and antibiotic therapy was prolonged and tailored according to microbiological culture findings.

### Outcomes

The primary outcome of the current study was the incidence of PCF. The secondary outcome was postoperative swallowing function. Swallowing was assessed three months after surgery using both subjective and objective measures. Subjective assessment was performed using the Eating Assessment Tool (EAT-10) questionnaire [18]. The validated Arabic version of the tool [19] was used, as all participants were Arabic-speaking. EAT-10 consists of ten items scored from 0 to 4, where 0 indicates no difficulty and 4 indicates a severe problem. The total score is calculated by summing the responses, with scores greater than 3 considered abnormal. Higher scores reflect greater severity of swallowing impairment.

Swallowing function was objectively assessed using video-fluoroscopy (VFS). Patients were asked to swallow liquid consistencies consisting of water mixed with barium in a 1:1 ratio, as well as solid materials coated with barium. Liquid boluses of 5 mL (spoon) and 20 mL (cup) were administered during continuous swallowing. For solid assessment, patients were instructed to chew a cookie prior to swallowing. The presence of post-swallow residue, strictures, diverticula, and cricopharyngeal spasm was systematically recorded.

### Statistical analysis

Statistical analysis was conducted using SPSS software version 26 (SPSS Inc., PASW Statistics for Windows, Chicago, IL, USA). Categorical variables were summarized as frequencies and percentages. Continuous variables were expressed as mean ± standard deviation for normally distributed data, with normality assessed using the Kolmogorov–Smirnov test. A p-value of less than 0.05 was considered statistically significant.

Comparisons of categorical variables between groups were performed using the Chi-square test, Fisher’s exact test, or Monte Carlo simulation, as appropriate. For continuous variables with non-normal distribution, differences between the two study groups were analyzed using the Mann–Whitney U test. The independent samples Student’s t-test was applied to compare normally distributed quantitative data between two groups.

## Results

A total of fifty patients were enrolled in this study. The baseline demographic and clinical features of the study cohort are presented in Table 1. All participants were male, with a mean age of 62.06 ± 6.87 years. There was no statistically significant difference in age between the two groups (*p* = 0.6). Similarly, no significant differences were observed between groups regarding smoking status (*p* = 0.49) or the distribution of medical comorbidities.

**Table 1:** Demographic and preoperative clinical data of the study participants

		Cervical fascia group (*n* = 25)	Control group (*n* = 25)	Test of significance
Age (years)		63.92 ± 7.30	60.20 ± 5.99	t = 1.97 *p* = 0.6
Smoking	**Non-smokers**	0 (0%)	2 (8.0%)	FET = 2.09
	**Smokers**	25 (100%)	23 (92.0%)	*P* = 0.490
Medical co-morbidities	**Diabetes mellitus**	6(24%)	8(36%)	χ^2^=0.857 *P* = 0.355
	**Hypertension**	13(52%)	17(68%)	χ^2^=1.33 *P* = 0.248
	**Ischemic heart disease**	1(4.0%)	0	FET = 1.02 *P* = 1.0
Tumor T stage	**T2**	1 (4%)	2 (8%)	Mc = 3.10 *P* = 0.376
	**T3**	24 (96%)	23 (92%)	
Tumor N stage	**N0**	12(48%)	13(52%)	Mc = 6.40 *P* = 0.171
	**N1**	5(20%)	2(8%)	
	**N2a**	2(8%)	0	
	**N2b**	6(24%)	7(28%)	
	**N2c**	0	3(12%)	
Preoperative Hemoglobin concentration (g/dl)		12.93 ± 1.31	13.32 ± 1.39	t = 1.02 *p* = 0.312
Preoperative serum Albumin (g/dl)		3.98 ± 0.26	3.97 ± 0.28	t = 0.158 *p* = 0.875
Previous radiotherapy		11(44%)	8(32%)	χ^2^=0.764 *P* = 0.382
Preoperative tracheostomy		12(48%)	11(44%)	χ^2^=0.081 *P* = 0.777

Data are presented as mean ± SD or number (%)

t = Student’s t-test; χ² = Chi-square test; FET = Fisher’s exact test; MC = Monte Carlo test

Tumor staging was distributed as follows: in the cervical fascia group, one patient had T2 disease and 24 patients had T3 disease. In the control group, two patients were staged as T2 and 23 as T3, with no statistically significant difference between groups (*p* = 0.376). Regarding nodal staging, the cervical fascia group included N0 (*n* = 12), N1 (*n* = 5), N2a (*n* = 2), and N2b (*n* = 6). In the control group, nodal stages were N0 (*n* = 13), N1 (*n* = 2), N2b (*n* = 7), and N2c (*n* = 3). These differences were not statistically significant (*p* = 0.171) (Table 1).

Moreover, preoperative variables that could influence postoperative healing and the incidence of PCF were evaluated and compared between the two groups. No significant differences were found in preoperative hemoglobin or albumin levels, nor in the incidence of prior neck irradiation or preoperative tracheotomy (Table 1).

Regarding the intraoperative findings and procedures (Table 2), neck dissection was performed in 23 of 25 patients in the cervical fascia group: unilateral in 22 cases and bilateral in one case. In the control group, neck dissection was unilateral in 20 patients and bilateral in 2 patients, with no statistically significant difference between groups (*p* = 0.821). Thyroidectomy was also comparable between groups: in the cervical fascia group, hemithyroidectomy was performed in 8 patients and total thyroidectomy in 6, whereas in the control group, hemithyroidectomy was performed in 5 patients and total thyroidectomy in 4 (*p* = 0.365). These differences were not statistically significant.

**Table 2:** Operative procedures among studied groups

		Cervical fascia group *N* = 25	Control group *N* = 25	Test of significance
Neck dissection	**Unilateral**	22(88%)	20(80%)	Mc = 0.917 *P* = 0.821
	**Bilateral**	1(4%)	2(8%)	
	**No neck dissection**	2(8%)	3(12%)	
Thyroidectomy	**Hemithyroidectomy**	8(32%)	5(20%)	χ^2^=2.02 *P* = 0.365
	**Total thyroidectomy**	6(24%)	4(16%)	
	**NO thyroidectomy**	11(44%)	16(64%)	

χ^2^=Chi-Square test, MC: Monte Carlo test *statistically significant, data expressed as number (%)

Table 3 summarizes the study outcomes and other reported complications. The incidence of pharyngocutaneous fistula was significantly lower in the cervical fascia group compared with the control group. PCF occurred in 2 of 25 patients (8%) in the cervical fascia group compared with 8 of 25 patients (32%) in the control group, corresponding to an absolute risk reduction of 24% (95% confidence interval: 5% to 43%; *p* = 0.034). All fistulae were detected clinically between the third and tenth day postoperatively (average 5 days).

**Table 3:** Study outcomes and other complications

		Cervical fascia group *N* = 25	Control group *N* = 25	Test of significance
Pharyngocutaneous fistula		2(8%)	8(32%)	χ^2^=4.50 *P* = 0.034*
Eating assessment tool (EAT)−10 Questionnaire score		4(0–11)	12(0–25)	Z = 3.18 *P* = 0.001*
Video Fluoroscopy	**Free**	16(64%)	8(32%)	MC = 6.49 *P* = 0.039*
	**Pharyngeal wall residue**	4(16%)	3(12%)	
	**Cricopharyngeal muscle spasm**	2(8%)	9(36%)	
	**Strictures**	0 (0%)	2 (8%)	
	**Diverticula**	3(12%)	3(12%)	
Other complications	**Fever**	5(20%)	6(24%)	χ^2^=0.117 *P* = 0.733
	**Hypocalcemia**	4(16%)	1(4%)	FET = 2.0 *P* = 0.157
	**Wound hematoma**	1 (4%)	0	FET = 1.02 *P* = 1.0
	**Chyle leak**	0	1(4%)	FET = 1.02 *P* = 1.0

Z: Mann Whitney U test, MC: Monte Carlo test, ꭓ^2^=Chi-Square test, FET: Fisher exact test, *statistically significant, data expressed as number (%) and as median (range) for questionnaire score

Spontaneous fistula healing was reported in the two patients in the cervical fascia group, and in 7 out of 8 patients in the control group. Healing occurred in average of 16 days (10–32). One patient in the control group required surgical closure of the fistula after failure of conservative treatment for 3 months.

Swallowing function, the secondary outcome of the study, was significantly better in the cervical fascia group. Subjective assessment using the EAT-10 demonstrated median scores of 4 (range: 0–11) in the cervical fascia group compared with 12 (range: 0–25) in the control group with a median difference of 6 points (95% confidence interval: 10 to 3; *p* = 0.001).

Objective evaluation using videofluoroscopy further supported the observed difference between groups. In the cervical fascia group, 16 patients (64%) demonstrated normal (free) swallowing, 4 patients (16%) showed pharyngeal wall residue, 2 patients (8%) exhibited cricopharyngeal muscle spasm, and 3 patients (12%) were found to have diverticula; no strictures were detected in this group. In contrast, the control group demonstrated normal swallowing in 8 patients (32%), pharyngeal wall residue in 3 patients (12%), cricopharyngeal muscle spasm in 9 patients (36%), strictures in 2 patients (8%), and diverticula in 3 patients (12%). A statistically significant difference was observed (*p* = 0.039) (Table 3).

Regarding other reported complications, fever was the most frequently observed (*n* = 11), followed by hypocalcemia (*n* = 5), chyle leak (*n* = 1), and wound hematoma (*n* = 1). There were no statistically significant difference between both groups in the incidence of these complications as shown in Table 3.

## Discussion

The use of the superiorly based cervical fascia flap for pharyngeal repair after total laryngectomy was first described by Zorlu et al. [17] in 2024. In their retrospective study, outcomes were evaluated in 22 patients who underwent cervical fascia flap reinforcement compared with 21 patients who did not. They reported a significantly lower incidence of pharyngocutaneous fistula in the fascia group compared with the control group. To our knowledge, the current study represents the first randomized clinical trial evaluating the efficacy of the cervical fascia flap in reducing the incidence of PCF after total laryngectomy.

In the current study, the overall incidence of PCF after total laryngectomy was 20% (10 of 50 patients). The incidence was substantially lower in the cervical fascia group (8%) compared with the control group (32%). Reported rates of PCF in the literature show considerable variability, ranging from less than 10% to more than 50% [6].

Fascia is a tissue characterized by relatively poor vascularity and low metabolic activity, yet it demonstrates resistance to adverse factors such as saliva and infection [20]. Zorlu et al. [17] reported that these properties are advantageous when fascia is applied as a separate reinforcing layer over the pharyngeal closure. In such cases, the integrity of the fascial layer is maintained and functions as an additional barrier, thereby supporting the healing process even in the presence of minor dehiscence of the underlying mucosal sutures.

Poutoglidis et al. [11] described a similar technique in which a layer of the muscular division of the pretracheal fascia investing the strap muscles was applied over the pharyngeal repair after total laryngectomy. They reported a marked reduction in the incidence of pharyngocutaneous fistula.

The use of cervical fascial flaps has also been reported in the literature for indications other than pharyngeal closure after total laryngectomy. Miličić [21] described a cervical fascia flap in hypopharyngeal tumors with laryngeal preservation. Élő et al. [20] also reported successful results with sternohyoid muscle fascia in partial vertical laryngectomy. In an animal study, they reported that epithelialization was completed on the fascia surface at 4 weeks postoperatively.

Numerous strategies have been explored to reduce the incidence of pharyngocutaneous fistula. Mechanical stapling devices have been utilized for pharyngeal closure with the aim of lowering fistula rates. Several studies have reported reduced rates of PCF with stapler-assisted suturing [22–24]. Conversely, Casasayas et al. [25] reported no significant impact of stapler use on PCF incidence. Moreover, the increased cost of stapling devices and their limited availability in routine otolaryngology practice represent important limitations to their widespread adoption.

Application of surgical sealants and biomaterials on the pharyngeal repair for enhancing healing has also been reported. The applied materials included albumin-polyaldehyde surgical sealant [26], platelet-rich fibrin [14], platelet-rich plasma [27], and bacterial cellulose [9] with reported outcomes showing variable results.

Several authors have advocated the use of myofascial flaps to reinforce pharyngeal repair to promote healing and reduce fistula rates. Reported techniques include the pectoralis major muscle flap [28], radial forearm or anterolateral thigh flap [16], sternomastoid muscle flap [29], infrahyoid myofascial flap [30] and temporoparietal fascia free flap [31]. These approaches have demonstrated effectiveness. However, their use is associated with prolonged operative time, additional skin incisions and tissue dissection, and an increased risk of donor-site morbidity.

In contrast to the aforementioned flaps, the cervical fascia flap used in the present study offers the advantage of being readily available within the same surgical field, without the need for additional incisions or exposure to donor-site morbidity. Moreover, it demonstrated a clear benefit in reinforcing the pharyngeal repair and, consequently, in reducing the incidence of pharyngocutaneous fistula.

Swallowing function outcomes in the present study were more favorable in the cervical fascia group than in the control group. This difference may be due to the higher incidence of cricopharyngeal spasm and stricture formation associated with the use of a muscular second layer of repair. Similar observations were reported by Poutoglidis et al. [11] who applied a fascial layer over the mucosal closure during pharyngeal repair without suturing the muscle layer and demonstrated favorable postoperative swallowing outcomes.

Additionally, Zorlu et al. [17] proposed that that the superiorly based fascia flap would facilitate swallowing with its elevation effect in the early postoperative period. Based on these findings, the cervical fascia appears to represent a more favorable option for second-layer reinforcement than the constrictor muscle. Its use enhances and strengthens the pharyngeal closure, resulting in improved healing, lower fistula rates, and better postoperative swallowing outcomes.

The authors of the present study hypothesize that cervical fascia may provide a more effective second layer of reinforcement than the constrictor muscle because the muscular layer is typically sutured in the midline, like the underlying pharyngeal mucosa. Consequently, apposition of both suture lines may predispose to simultaneous disruption in the event of dehiscence or salivary leakage. In contrast, the cervical fascia functions as a single reinforcing layer that is sutured laterally on both sides, thereby providing more reliable support to the underlying closure. This effect is further enhanced by the intrinsic properties of fascia, including its low metabolic demand and relative resistance to infection.

To ensure oncologic safety, the cervical fascia flap should be applied only in appropriately selected cases. Zorlu et al. [17] avoided using the fascia flap in the presence of strap muscle invasion. Similarly, in the present study, patients with extralaryngeal extension (T4a disease) were excluded. In addition, the lateral border of the sternohyoid muscle defines the lateral limit of the flap. The investing fascia covering levels II, III, and IV lymph node basins is not incorporated into the flap and is excised en bloc with the lateral neck dissection specimen.

The present study has several limitations. Thes include the lack of multivariate adjustment for potential confounders known to influence the risk of pharyngocutaneous fistula, including neck dissection, thyroidectomy, prior radiotherapy, preoperative tracheostomy, and nutritional status. Owing to the relatively small sample size and limited number of fistula events, multivariate analysis was not feasible. Larger, adequately powered studies are required to confirm the independent effect of cervical fascia reinforcement. Additionally, the follow-up period for swallowing outcomes was relatively short. Therefore, longer follow-up studies are required to assess long-term functional results. Finally, multiple statistical comparisons represent a limitation of this study.

The external validity of the present study is subject to several limitations. All included patients were male, reflecting the demographic characteristics of laryngeal carcinoma in the studied population. In addition, this was a single-center study, and all procedures were performed by a single, experienced surgical team using a standardized technique. Multicenter studies involving more diverse patient populations and multiple surgical teams are warranted to confirm the generalizability of these findings.

In addition, future research should explore the expansion of indications to include patients with extralaryngeal extension, while incorporating long-term oncological outcomes to ensure that the use of the cervical fascia flap in such populations remains oncologically safe.

## Conclusion

The use of cervical fascia for pharyngeal repair after total laryngectomy is effective in reducing the incidence of pharyngocutaneous fistula. Its main advantages include technical simplicity, cost-effectiveness, and availability within the same surgical field, without the need for additional incisions, prolonged operative time, or donor-site morbidity. Furthermore, this technique is associated with favorable postoperative swallowing outcomes.

## References

[1] Tawfik A, El-Fattah AMA, Hassan A, Helal FA, Ebada HA (2024) Discrepancy between clinical and pathological staging of laryngeal carcinoma: a dilemma to be solved. Eur Arch Otorhinolaryngol 281(5):2507–2513 38345614 10.1007/s00405-024-08506-2 PMC11023994

[2] Salem EH, Habaza FR, Ebada HA, Abu Shady EF, Elkotamy SN, Thabet AH, Abdelmeguid AS, Kamal E, Hamza A, Abdelaziz M (2025) Lymph node yield/ratio, neutrophil-lymphocyte ratio: prognostic factors in cN0 laryngeal carcinoma. Laryngoscope 135(6):2037–2043 39754398 10.1002/lary.31986

[3] Cömert E, Tunçel Ü, Torun MT, Kiliç C, Cengiz AB, Kaya M (2014) Pectoralis major myofascial flap in salvage laryngectomy. J Laryngol Otol 128(8):714–719 25026463 10.1017/S0022215114001479

[4] Tsetsos N, Poutoglidis A, Vlachtsis K, Stavrakas M, Nikolaou A, Fyrmpas G (2021) Twenty-year experience with salvage total laryngectomy: lessons learned. J Laryngol Otol 135(8):729–736 34219631 10.1017/S0022215121001687

[5] Gendreau–Lefèvre AK, Audet N, Maltais S, Thuot F (2015) Prophylactic pectoralis major muscle flap in prevention of pharyngocutaneous fistula in total laryngectomy after radiotherapy. Head Neck 37(9):1233–1238 24801433 10.1002/hed.23742

[6] Paydarfar JA, Birkmeyer NJ (2006) Complications in head and neck surgery: a meta-analysis of postlaryngectomy pharyngocutaneous fistula. Arch Otolaryngol Head Neck Surg 132(1):67–72 16415432 10.1001/archotol.132.1.67

[7] Mattioli F, Bettini M, Molteni G, Piccinini A, Valoriani F, Gabriele S, Presutti L (2015) Analysis of risk factors for pharyngocutaneous fistula after total laryngectomy with particular focus on nutritional status. Acta Otorhinolaryngol Ital 35(4):243–248 26824210 PMC4731886

[8] Dedivitis RA, Aires FT, Cernea CR, Brandao LG (2015) Pharyngocutaneous fistula after total laryngectomy: systematic review of risk factors. Head Neck 37(11):1691–1697 24958209 10.1002/hed.23804

[9] Demir B, Sarı M, Binnetoglu A, Yumusakhuylu AC, Filinte D, Tekin İÖ, Bağlam T, Batman AÇ (2018) Comparison of pharyngocutaneous fistula closure with and without bacterial cellulose in a rat model. Auris Nasus Larynx 45(2):301–305 28487041 10.1016/j.anl.2017.04.005

[10] Calli C, Pinar E, Oncel S (2011) Pharyngocutaneous fistula after total laryngectomy: less common with mechanical stapler closure. Ann Otol Rhinol Laryngol 120(5):339–344 21675591 10.1177/000348941112000510

[11] Poutoglidis A, Forozidou E, Fyrmpas G, Mantsopoulos K, Paraskevas GK, Lazaridis N, Savvakis S, Karamitsou P (2024) A novel and simple technique to reduce the rate of pharyngocutaneous fistula formation following a total laryngectomy-our initial results. Indian J Otolaryngol Head Neck Surg 76(1):997–1001 38440597 10.1007/s12070-023-04343-7 PMC10908975

[12] Mandor EA, Ebada HA, El–Sisi HE, Abd El-Fattah AM, Tawfik A (2025) Stapler-assisted total laryngectomy: surgical techniques and outcomes. Egypt J Otolaryngol 41(1):210

[13] Tokat T, Muderris T, Aysel A, Sari E, Erol F (2021) The efficiency of polyglycolic acid felt in the prevention of pharyngocutaneous fistula after total laryngectomy. Am J Otolaryngol 42(6):103164 34352675 10.1016/j.amjoto.2021.103164

[14] Eid AM, Ebada HA, El-Fattah AMA, Tawfik A (2021) Platelet-rich fibrin: an autologous biomaterial for healing assistance of pharyngeal repair in total laryngectomy. Eur Arch Otorhinolaryngol 278(2):463–470 33009930 10.1007/s00405-020-06404-x

[15] Patel UA, Keni SP (2009) Pectoralis myofascial flap during salvage laryngectomy prevents pharyngocutaneous fistula. Otolaryngol Head Neck Surg 141(2):190–195 19643250 10.1016/j.otohns.2009.03.024

[16] Fung K, Teknos TN, Vandenberg CD, Lyden TH, Bradford CR, Hogikyan ND, Kim J, Prince ME, Wolf GT, Chepeha DB (2007) Prevention of wound complications following salvage laryngectomy using free vascularized tissue. Head Neck: J Sci Specialties Head Neck 29(5):425–430 10.1002/hed.20492 17274047

[17] Zorlu ME, Kertmen C, Aysel A, Yilmaz F, Dalgic A, Muderris T (2024) Use of cervical fascia to prevent pharyngocutaneus fistula after total laryngectomy. Laryngoscope 134(12):4964–4970 38940495 10.1002/lary.31606

[18] Belafsky PC, Mouadeb DA, Rees CJ, Pryor JC, Postma GN, Allen J, Leonard RJ (2008) Validity and reliability of the Eating Assessment Tool (EAT-10). Annals of Otology. Rhinology Laryngology 117(12):919–924 10.1177/000348940811701210 19140539

[19] Farahat M, Mesallam TA (2016) Validation and cultural adaptation of the Arabic version of the Eating Assessment Tool (EAT-10). Folia Phoniatr Logop 67(5):231–237 10.1159/000442199 26844779

[20] Élő J, Horváth E, Késmárszky R (2000) A new method for reconstruction of the larynx after vertical partial resections. Eur Arch Otorhinolaryngol 257(4):212–215 10867836 10.1007/s004050050224

[21] Miličić D (2003) The use of cervical fascia for hypopharyngeal reconstruction with laryngeal preservation. Eur Arch Otorhinolaryngol. 10.1007/s00405-002-0550-2 12709804 10.1007/s00405-002-0550-2

[22] Zhang X, Liu Z, Li Q, Liu X, Li H, Liu W, Li Q, Guo Z, Zeng Z (2013) Using a linear stapler for pharyngeal closure in total laryngectomy. Eur Arch Otorhinolaryngol 270(4):1467–1471 22986414 10.1007/s00405-012-2180-7

[23] Ismi O, Unal M, Vayisoglu Y, Yesilova M, Helvaci I, Gorur K, Ozcan C (2017) Stapler esophageal closure during total laryngectomy. J Craniofac Surg 28(1):e35–e40 27893554 10.1097/SCS.0000000000003196

[24] Mandor EA, Ebada HA, El-Fattah AMA, Kamal E, Baz H, Tawfik A (2024) Stapler versus conventional pharyngeal repair after total laryngectomy: a randomized clinical trial. Eur Arch Otorhinolaryngol 281(8):4273–4280 38739184 10.1007/s00405-024-08696-9 PMC11266385

[25] Casasayas M, Sansa A, García-Lorenzo J, López M, Orús C, Peláez X, Quer M, León X (2019) Pharyngocutaneous fistula after total laryngectomy: multivariate analysis of risk factors and a severity-based classification proposal. Eur Arch Otorhinolaryngol 276(1):143–151 30426230 10.1007/s00405-018-5200-4

[26] Stephenson KA, Pandey S, Lubbe DE, Fagan JJ (2018) Use of surgical sealant in the prevention of pharyngocutaneous fistula after total laryngectomy. Head Neck 40(12):2606–2611 30488504 10.1002/hed.25334

[27] Eryılmaz A, Demirci B, Gunel C, Doger FK, Yukselen O, Omurlu IK, Basal Y, Agdas F, Basak S (2016) Can tissue adhesives and platelet-rich plasma prevent pharyngocutaneous fistula formation? Auris Nasus Larynx 43(1):62–67 26229017 10.1016/j.anl.2015.06.012

[28] Smith TJ, Burrage KJ, Ganguly P, Kirby S, Drover C (2003) Prevention of postlaryngectomy pharyngocutaneous fistula: the Memorial University experience. J Otolaryngol 32(4):222–225 14587560 10.2310/7070.2003.41697

[29] Ibrahim SG, Wahba BM, Elbatawi AM, Eltelety AM (2017) Sternocleidomastoid flap augmentation of the pharyngeal closure after total laryngectomy. Eur Arch Otorhinolaryngol 274(8):3197–3202 28508179 10.1007/s00405-017-4582-z

[30] Kadota H, Fukushima J, Kamizono K, Masuda M, Tanaka S, Yoshida T, Nakashima T, Komune S (2013) A minimally invasive method to prevent postlaryngectomy major pharyngocutaneous fistula using infrahyoid myofascial flap. J Plast Reconstr Aesthet Surg 66(7):906–911 23615183 10.1016/j.bjps.2013.03.033

[31] Higgins KM, Ashford B, Erovic BM, Yoo J, Enepekides DJ (2012) Temporoparietal fascia free flap for pharyngeal coverage after salvage total laryngectomy. Laryngoscope 122(3):523–527 22147652 10.1002/lary.22477

